# Improving prediction of COVID-19 evolution by fusing epidemiological and mobility data

**DOI:** 10.1038/s41598-021-94696-2

**Published:** 2021-07-26

**Authors:** Santi García-Cremades, Juan Morales-García, Rocío Hernández-Sanjaime, Raquel Martínez-España, Andrés Bueno-Crespo, Enrique Hernández-Orallo, José J. López-Espín, José M. Cecilia

**Affiliations:** 1 Center of Operations Research, Miguel Hernandez University of Elche (UMH), 03202 Elche, Spain; 2 Computer Science Department, Universidad Católica de Murcia (UCAM), 30107 Murcia, Spain; 3 Computer Engineering Department, Univesitat Politècnica de València (UPV), 46022 Valencia, Spain

**Keywords:** Applied mathematics, Computational science, Computer science, Scientific data, Statistics

## Abstract

We are witnessing the dramatic consequences of the COVID-19 pandemic which, unfortunately, go beyond the impact on the health system. Until herd immunity is achieved with vaccines, the only available mechanisms for controlling the pandemic are quarantines, perimeter closures and social distancing with the aim of reducing mobility. Governments only apply these measures for a reduced period, since they involve the closure of economic activities such as tourism, cultural activities, or nightlife. The main criterion for establishing these measures and planning socioeconomic subsidies is the evolution of infections. However, the collapse of the health system and the unpredictability of human behavior, among others, make it difficult to predict this evolution in the short to medium term. This article evaluates different models for the early prediction of the evolution of the COVID-19 pandemic to create a decision support system for policy-makers. We consider a wide branch of models including artificial neural networks such as LSTM and GRU and statistically based models such as autoregressive (AR) or ARIMA. Moreover, several consensus strategies to ensemble all models into one system are proposed to obtain better results in this uncertain environment. Finally, a multivariate model that includes mobility data provided by Google is proposed to better forecast trend changes in the 14-day CI. A real case study in Spain is evaluated, providing very accurate results for the prediction of 14-day CI in scenarios with and without trend changes, reaching 0.93 $R^2$, 4.16 RMSE and 1.08 MAE.

The COVID-19 pandemic is the biggest global challenge in our recent history, which puts the welfare state of today’s society at risk. Spain is undoubtedly among the countries most affected by the pandemic, with up to 3,697,987 total cases of infection, and a total of 80,196 deaths (as reported on June 7, 2021)^1^. Governments worldwide are taking drastic measures such as social distancing, contact tracing, perimeter closures and even quarantines, which are either reinforced or alleviated depending on the epidemiological status of the disease^2^. These non-sanitary measures focus on the reduction of human mobility, which has an important socio-economic effect^3^. For instance, according to the European Commission, the economic forecast for Spain is the worst in its recent history with a 9.4% drop in GDP, and an expected unemployment of up to 18.9% at the end of 2020. Globally speaking, the Organisation for Economic Co-operation and Development (OECD)^4^ stated that these bad economic projections will lead to widespread poverty, child malnutrition, stress, and suicides, just to mention a few of the dramatic consequences for the population . However, beyond the economic consequences, the measures of social distancing and lockdowns can raise new social scenarios in fundamental aspects such as education, gender violence, immigration and other new issues that may arise because of such extreme public health measures.

Early understanding of the evolution of the pandemic prevents scenarios that could increase the number of COVID-19 victims. Governments have implemented public health surveillance systems for COVID-19 based on the fundamental principles provided by the World Health Organization (WHO); i.e., tracking clinical and epidemiological figures such as confirmed, death, active cases, just to mention a few^5,6^. This information is usually provided by governments daily, and currently, these surveillance systems provide robust and stable information on the evolution of the pandemic^7^. However, this epidemiological information shows a posterior picture of the pandemic, i.e., once people have been infected and are showing symptoms, usually after an incubation period of 7-10 days^8^. From these epidemiological data, novel Machine learning (ML), Artificial Intelligence (AI) and data science methods can provide significant outcomes for tracking and detecting COVID-19 evolution at national and regional level^9^. All in all, the infection curve can be seen as a time series in which trend changes are hardly predictable, as it does not follow a seasonal pattern, mainly due to the chaotic interaction of people.

Figure 3 shows the 14-day CI in Spain from July 20, 2020 until January 2021. The first Spanish wave officially ended on July 20, 2020 and the 14-day CI started to increase again from that date onwards. It is worth mentioning that from the second wave until today, there have been several waves, understood as trend changes in the 14-days CI. At the beginning of October, 9th the 14-day CI started to increase again, matching with a vacation period at the national level, from October 9th to 12th. In addition, in mid-December a trend change of the 14-day CI was reported, also coinciding with a vacation period (December 8–12, 2020), which is increasing from that date until now. These trend changes are one of the most difficult scenarios for modelling. The 14-day CI is a time series that includes daily data from July. Besides, not every day is reported, COVID-19 data in Spain is only reported on working days, i.e., Monday through Friday, except holidays. The lack of historical data, as well as the scarce changes in trends during the training period makes it very difficult to let the models learn these changes.

In this paper, we propose a multivariate model to predict trend changes in the 14-day Cumulative Incidence (CI) of COVID-19. We conducted a comprehensive analysis of different mobility components offered by Google to incorporate this information into our multivariate model as exogenous information. The multivariate model resulting from adding this information can predict trend changes in 14-day CI with greater accuracy. The main contributions of the paper are the following:

1. Several state-of-the-art ML and statistical methods are evaluated to predict the 14-day CI, using only the historical information of this variable as input for two different scenarios, i.e., 14-day CI with trend changes and without trend changes in the time series.
2. A ensemble strategy is provided to combine previous models and provide an optimal prediction. These methods offer very good performance for this time series when there are no clear trend changes.
3. A multivariate model is designed and fed with 14-day CI and mobility variables provided by Google as exogenous information.
4. The multivariate model is optimized using operational research techniques to achieve better prediction of trend changes in 14-day CI.
5. The evaluation is based on information from several waves in Spain in which clear trend changes were reported.

The reminder of the paper is structured as follows. Firstly, we discuss the related work. Then, the methods of this article are introduced in “Methods” section, including the main ML and statistical models proposed, their ensemble and the exogenous information targeted. Finally, “Evaluation and results” section shows the main results and finding of our article before the main conclusions and directions for future work are introduced.

## Related work

Since the right beginning of the COVID-19, scientists have struggled on designing models that could forecast not only the evolution of the disease but also the impact of the different measures taken. The problem is that these models must characterise not only how the virus spread, which is far from being understood, but also about human behaviour, which can be erratic. Firstly, it is necessary to evaluate and model how fast the COVID-19 is spreading. A fundamental epidemiological quantity, the reproductive number *R*, represents the average number of new infections an infected person can generate (so the greater the number, the faster the spreading). First estimations of the $R_0$ value for the COVID-19 evidenced a relatively high value, in the range (2.4–5.6)^10–12^. Fortunately, measures such as social distancing, facial masks and mobility reduction have allowed health authorities to control the spread of the disease.

Different types of models have been proposed for forecasting COVID-19 evolution: compartmental models, statistical-based models and machine learning (ML) based models^13^. In epidemiological compartmental models, the population is assigned to different compartments (for example, the simple SIR models with three compartments: Susceptible, Infectious, and Recovered). These compartmental models have been used to evaluate and forecast the impact of the different measures taken, such as quarantine, isolation and contact tracing. For example, in^14,15^ the authors model and evaluate the general effects of containment mechanisms. Regarding contact tracing, in^10,11^ it was stated that contact tracing and isolation as currently practiced is not helping in preventing the COVID-19 pandemic. Finally, in^16,17^, the authors evaluated the impact of the technological aspects (such as resolution, centralised vs decentralised approaches) of the current smart-based contact tracing application showing that for being effective, it would have required a high adoption rate and a centralised technology. Unfortunately, it was not the case, so these kinds of contact tracing applications failed to control the disease.

On the other hand, statistical-based models, i.e., time series analysis and forecasting, only rely on past data to predict the near future. There are many different methods, such as Auto-Regressive Moving Average (ARMA), Auto-Regressive Integrated Moving Average (ARIMA), Support Vector Regressor (SVR), Linear Regressor polynomial (LRP), Bayesian Ridge Regression (BRR), Linear Regression (LR), Random Forest Regressor (RFR), Holt-Winter Exponential Smoothing (HW), and Extreme Gradient Boost Regressor (XGB). Note that some authors consider some of these methods as Machine Learning Methods^18^ but none of them seems to improve the overall quality of the prediction^19–21^ (see below for a detailed description of this references). Among these models, we may highlight ARIMA model^22^, which has shown good results forecasting the COVID-19 infections. For instance, Benvenuto et al.^23^ proposed the use of ARIMA models to predict the COVID-19 spread around the world, while Perone et al.^24^ proposed a model for different regions of Italy and Sahai et al.^25^ did the same for the top five affected countries. Nevertheless, these models can only predict short-time behaviour as intervals of confidence grows extremely fast as time elapses^26^. Petropoulos et al.^27^ also recognized the limitations of forecasting longer term trajectories of an outbreak.

As previously commented, some authors consider most of the previous statistical methods to be part of more general Machine learning (ML) and Deep Learning (DL) methods^19^. For example, Shahit et al.^28^ used DL methods for the prediction of time series of confirmed cases, deaths and recoveries in COVID-19 affected countries, where the performance of models was measured by mean absolute error (MAE), root mean square error (RMSE) and $\hbox {R}^2$. They focus on different variables (but not 14-day CI) but with stable trends. Similarly, Zerorual et al.^29^ compared up to five DL models for COVID-19 forecasting using different COVID-19 information including, Italy, Spain, France, China, USA and Australia. Nevertheless, more specific ML methods such as neural networks and Support Vector Machines (SVMs) have shown to perform poorly since they require more training data than the currently available datasets^20,21^. Furthermore, as stated by Ribeiro et al.^30^ this fact can also be attributed to the chaotic dynamics of the analysed data, as well as the diversity of exogenous factors.

Several studies have shown the relationship between mobility and the disease spread. Linka et al.^31^ showed a strong correlation between the reduction in mobility and the effective reproduction number across Europe, which was particularly high for countries such as the Netherlands, Germany, Ireland, Spain, and Sweden (which have a Spearman’s rank correlation $\rho$ of 0.99). The authors in^32,33^ found that mobility statistics offered in open COVID-19 datasets showed the evolution of the COVID-19 spread in China, placing the contagious peak at the early beginning of 2020. A recent study using mobile phone data of more than 13 million users in Spain^34^, has shown that these data can be used as a predictor of COVID-19-related deaths. Particularly, they stated that there is a critical level (around 70% of the radius of gyration, which quantifies the mobility range of an individual during a given week^35^) when hospitalizations and deaths tend to increase two to three weeks after this threshold is exceeded. Finally, Google and Apple mobility data, which are used in this paper, has demonstrated to be of great help in quantifying and predicting the effects of COVID-19. For example, Cot et al.^36^ quantify the effects of social distancing on the COVID-19 spreading dynamics in Europe and in the USA, and Nouvellet et al.^37^ show the correlation between the reduction in mobility and COVID-19 transmission. One key aspect of all these models is the quality of the data used. Having a wide range of data, updated on a real-time basis and accessible is critical to characterizing disease outbreaks and obtaining useful models^38^. Nevertheless, better data are necessary, but not sufficient. As stated by Castro et al.^26^, human models are really hard to model since there is always an uncertainty in human behaviour, so most models can fail to forecast some important issues such as turning points and the end of the expansion.

Summing up, the problem with the described forecasting models is to accurately predict trend changes (i.e., waves) when using only previous historical information. These changes in trends can depend on varying external elements, such as mobility, social distancing, etc. Therefore, a way to improve the precision of the previous forecasting methods is to combine several data sources. Particularly, in this paper, we show that the utilisation of mobility data can improve forecasting when only time series (such as 14-day CI) are used.

## Methods

Temporary data are omnipresent in many application domains, such as medicine, agriculture or robotics^39,40^. Increasingly, time series forecasting is being introduced in these fields which follows a quantitative approach that uses historical information along with certain associated patterns such as trends, seasonality and irregular components to predict future observations. Trend data in the time series offers long-term information for the prediction. Seasonality are patterns in the time series that occur at specific and regular intervals. Finally, irregular components are unsystematic fluctuations due to external factors. Having access to historical time-series data, forecasting models can be used to understand the behaviour of the time series. However, the irregular components of the time series are difficult to predict as they do not follow a given pattern. Generally speaking, time-series models cannot learn these irregular components from the historical data of the time series, so they need additional information to identify these possible events^41^

Indeed, the evolution of the 14-day CI of COVID-19 is based on irregular components that are mainly caused by the different implementations of the national legislation that reduces people’s mobility^42^. Several ICT companies such as Google or Apple have provided mobility data taken from smartphones that run mobility applications, such as Google Maps or Maps from Apple Maps, to figure out the changes that have occurred in people’s mobility as a result of the policies to deal with COVID-19^43^. As previously explained in “Related work” section, several works have been recently done to predict the COVID-19 evolution based on trends and seasonality in time series, but none of them has not analysed trend changes due to these irregular components. This section introduces the ML and statistical univariate models used in this article to predict the 14-day CI using only the endogenous variable; i.e. previous observations of the 14-day CI. These models are combined through an ensemble approach that uses different consensus strategies based on quality metrics that are first described. Finally, the multivariate model is introduced to improve the prediction of the 14-day CI, in those time lags where there are trend changes.

### Metrics and statistical models used

The main metrics and statistical models used in this work are the following (where $x_i$ is the real data for instance *i* and $P_i$ is the prediction for instance *i*):

- *Coefficient of determination* ($\hbox {R}^2$) is used to analyse how differences in one variable can be explained by differences in a second variable. It is a value ranging from 0 to 1 and indicates that the regression line represents none or all of the data, respectively, so that the higher the value, the better the goodness of fit of the model^44^.
  1 $$\begin{aligned} R^2=\frac{( \sum _{i=1}^{n}{(x_i-\bar{x})(P_{i}-\bar{P})})^2}{\sum _{i=1}^{n}{(x_i-\bar{x})^2}\sum _{i=1}^{n}{(P_{i}-\bar{P})^2}} \end{aligned}$$
- *Root mean square error (RMSE)* is the standard deviation of the prediction errors, which are a measure of the distance of the data from the regression line, indicating the concentration of the data around the line of best fit. It is, therefore, a measure of the dispersion of these errors (also known as residuals)^45^.
  2 $$\begin{aligned} RMSE=\sqrt{\frac{\sum _{i=1}^{n}(x_i-P_{i})^2}{n}} \end{aligned}$$
- *Mean absolute error(MAE)* allows measurement of the average magnitude of the errors for a set of predictions, regardless of their direction. It represents the mean of the absolute differences in the sample between the prediction and the actual observation, taking into account that all individual differences are of equal significance^45^.
  3 $$\begin{aligned} MAE=\frac{\sum _{i=1}^{n}|x_i-P_{i}|}{n} \end{aligned}$$
- *Spearman correlation* Spearman’s correlation coefficient is a non-parametric measure of rank correlation; i.e. statistical dependence of the ranking between two variables. It measures the strength and direction of the association between two ranked variables^46^.
- *Granger causality* Granger causality is a testing framework comparing the unrestricted model, in which a time series *y* is explained by the lags of *y* and the lags of an additional series of observations *x* (both lags up to the same fixed order), and the restricted model, in which *y* is only explained by the lags of *y*. Thus, Granger causality determines if one time series is helpful for predicting another, and in some cases, it may be used to assert stronger causal statements^47^.
- *Principal component analysis (PCA)* The aim of this technique is to reduce the dimensionality of multivariate data preserving as much of the relevant information as possible^48^.

### Ensemble approach for univariate prediction

**Figure 1:**
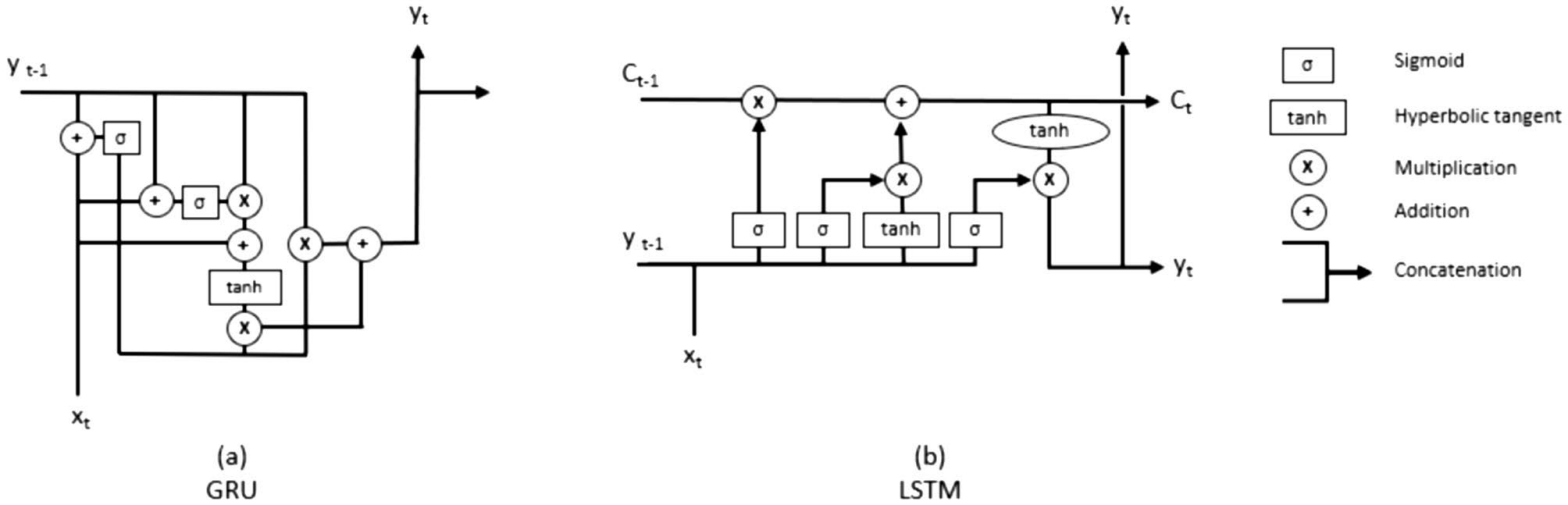
Diagram of a GRU and LSTM unit. Where $x_t$ represents the input and $y_t$ the forecast in a step ($y_{t-1}$ for forecast in the previous steps). For LSTM, the $C_t$ indicates the state that is passing from one LSTM unit to another.

This subsection proposes a combination of time series and ML models and techniques to provide a consensus strategy that brings all the results into one. Each method and model has demonstrated in the literature good results for predicting different epidemiological variables related to COVID-19. Moreover, different configurations and/or parameterisations of these models are also important for the quality of the predicted results. With the proposed ensemble, the search space of the models is explored automatically in order to obtain the best possible prediction. The statistical and machine learning methods under study are the following:

1. Autoregresive (AR) is a univariate model^49^ where a prediction is made using a linear combination of past values of that variable. The term autoregression indicates that it is a regression of the variable against itself. Thus, an autoregressive model is established according to its order *p*. Autoregressive models are remarkably flexible to handle a wide range of different time series patterns.
2. Autoregressive Integrated Moving Average (ARIMA) is a linear statistical model^50^, which uses variations and regressions of statistical data in order to find patterns for a prediction into the future. Automatic Regression (AR) is the term that refers to the delays of the differentiated series ($T-i$), Moving Average (MA) refers to the delays of the errors and integration (I) is the number of differences used to make the time series stationary.
3. Long short-term memory (LSTM) is a type of recurrent neural architecture with a state memory and multilayer cell structure^51^. LSTM unit is composed of a cell, an input gate, an output gate and a forget gate. The cell remembers values over arbitrary time intervals and the three gates regulate the flow of information into and out of the cell(Fig. 1b). The LSTM differs from a classic recurrent network in that it does not overwrite its content at each time step but is able to decide whether to keep the existing memory through the introduced doors. If the LSTM unit detects an important characteristic of an input sequence at an early stage, it carries this information over long distances, therefore it detects long-distance dependencies.
4. Gate Recurrent Unit (GRU) is a type of recurrent neural network, which presents a modification, which allows to solve a problem of this type of recurrent networks which is the vanishing gradient problem since the model is not washing out the new input every single time but keeps the relevant information and passes it down to the next time steps of the network^52^. It is similar to LSTM but without memory cells, which makes them simpler to compute and implement. It is composed of two gates (reset and update) (Fig. 1a), so that it allows each recurrent unit to capture the dependencies in an adaptive way in different time scales. Through these two gates, it is decided what information should be passed on at the output, without eliminating information that is apparently irrelevant to the prediction, so that the information is retained for a long time.

In the process of combining the information of the proposed ensemble approach, the validation metrics for the regression task are used. Particularly, our ensemble approach uses the coefficient of determination ($\hbox {R}^2$), root mean square error (RMSE) and mean absolute error (MAE) metrics^53^. Before describing in detail the phases of this proposed ensemble approach, the 4 combination methods used to obtain and calculate the model for the inference are described. The combination methods used are briefly detailed below:

- *Maximum*The predictions of the model that has a metric greater than $\hbox {R}^2$ are selected.
- *Minimum* The models with the lowest RMSE and MAE metrics are selected and a weighted average is computed.
- *Average* An average of all models is made without taking into account their values.
- *Weighted average* A weighted average is made based on the $\hbox {R}^2$ score of each model.

**Figure 2:**
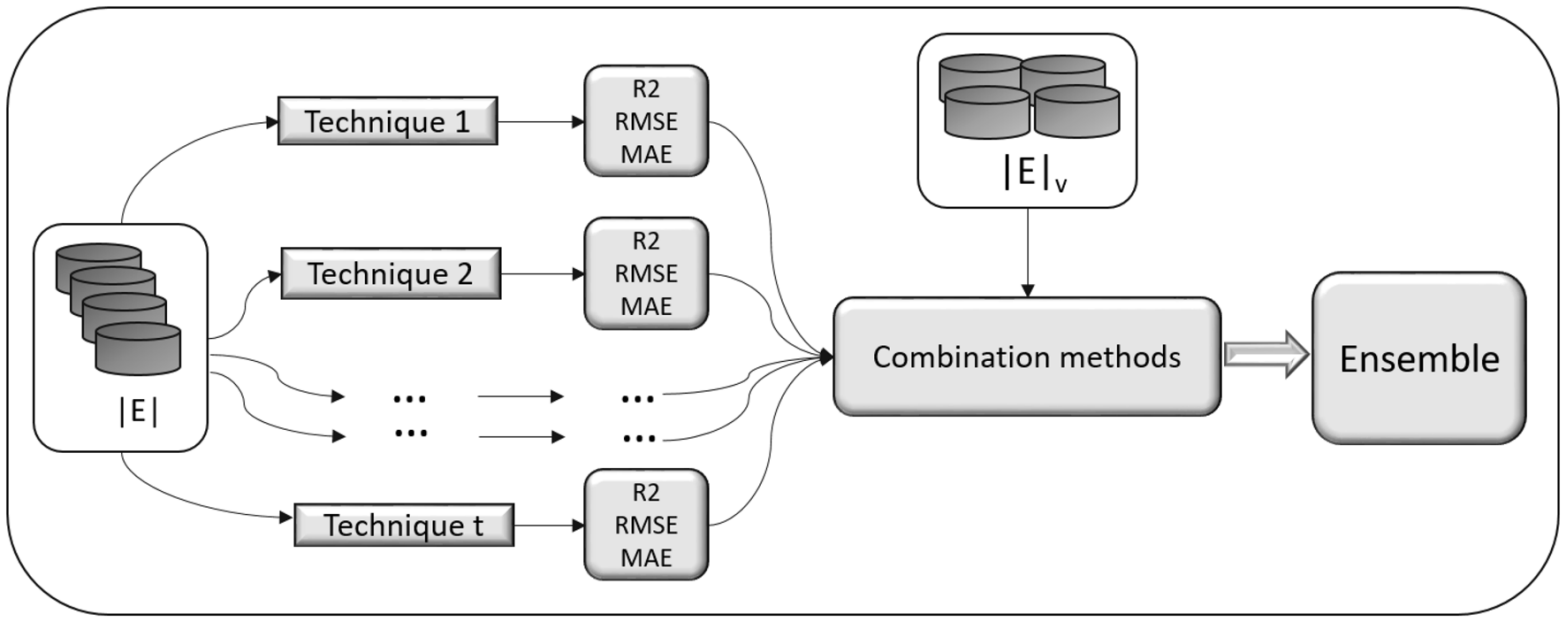
Outline of the proposed ensemble approach.

The proposed ensemble approach consists of the following steps. Figure 2 summarizes these steps.

1. Let’s be |*E*| , the training dataset and $|E|_v$, a validation dataset.
2. Each technique *t* is trained with the |*E*| dataset, generating $\hbox {P}_{|E|}$ for each *t*.
3. For each technique *t*, the values $R^2$, RMSE and MAE are calculated using the predictions $P_{|E|}^t$ and $|E|_v$ dataset.
4. Using the combination methods |*C*|, models whose predictions are effective are selected.
5. Depending on the combination method, the $P_{|E_v|}$ predictions are calculated by taking the data from the validation dataset $|E|_{v}$ as input.
6. The metrics of $R^2$, RMSE and MAE are calculated with the predictions $P_{|E|_v}$, leaving the model built and ready to infer values.
7. Equation (4) is used to infer a new value *i* in the model:
  4 $$\begin{aligned} P_{i} = \frac{P_i^{MaxR_t}+P_i^{MinRMSE_t}+P_i^{MinMAE_t}}{3} \end{aligned}$$
  where $P_i^{MaxR_t}$ is the prediction for instance *i* that provides the *t* model with the maximum $R^2$; $P_i^{MinRMSE_t}$ is the prediction for instance *i* that provides the *t* model with the minimum RMSE and $P_i^{MinMAE_t}$ is the prediction for instance *i* that provides the *t* model with the minimum MAE.

### Measuring mobility for the multivariate model

Reducing mobility has been one of the main tools that all governments worldwide are using to prevent the COVID-19 spread. Tracing infection from mobility data has been used from the early beginning of the COVID-19 outbreak. Kraemer et al.^32,33^ found that mobility statistics offered in open COVID-19 datasets showed the evolution of the COVID-19 spread in China, placing the contagious peak at the early beginning of 2020. Therefore, the measurement of mobility in different cities has been subjected to study by different public and private organizations. Huang et al.^54^ showed that mobility patterns obtained from Twitter can quantitatively reflect the mobility dynamics.

Google mobility data (GMD) (https://www.google.com/covid19/mobility/) is a tool developed by Google to deal with the COVID-19. It shows a set of aggregated and anonymized data obtained from information in products such as Google Maps^55^. This data is provided through local mobility reports which offer valuable information on changes in people’s mobility patterns as a consequence of the measures taken by the governments to deal with the COVID-19 pandemic. Among the information found in these reports, of particular interest to us are the movement trends of citizens over time. This information is arranged by geographical area and classified into various categories of places, such as workplaces, stores, supermarkets, leisure spaces, pharmacies, parks, transportation stations and residential areas. The main variables GMD provides are the following:

- *Retail and recreation* This variable shows mobility trends for places such as restaurants, cafes, museums, malls, cinemas and libraries.
- *Supermarket and pharmacy* This variable shows mobility trends for places such as supermarkets, food warehouses and pharmacies.
- *Parks* This variable show mobility trends for places such as national parks, public beaches, plazas and public gardens.
- *Public transport* This variable shows mobility trends for places that are public transport hubs, such as train stations, subway or bus.
- *Workplaces* This variable shows mobility trends for places of work.
- *Residential* This variable shows mobility trends for places of residence.

The number provided by GMD is used to compare the mobility on the date of the report with the mobility on the day of the reference value. The data corresponding to the date of the report is calculated (if the information is available) and a positive or negative percentage is shown. The data shows how the number of visitors to (or time spent in) the categorized locations changes compared to our baseline. A baseline represents a normal value on that day of the week. The baseline is the average value for the 5-week period from January 3 to February 6, 2020. In each region-category, the baseline is not a single value, but 7 individual values. The same number of visitors on two different days of the week results in different percentage changes. It is important to note that baseline days never change. In the calculation of the reference values, the seasonality has not been taken into account. For example, the number of people going to the parks usually increases as the weather improves.

A multivariate model including these variables is proposed to predict 14-day CI. Our first approach was to explore a multivariate regression model which includes the ensemble information and additional information in the mobility variables as exogenous information. The multivariate equation is shown in Eq. (5).

5 $$\begin{aligned} CI_{14-day} = \beta _0 + \beta _1 (Ensemble) + \beta _2 GMD_2 + \beta _3 GMD_3 +\cdots + \beta _i GMD_4 \end{aligned}$$

where the response variable is $CI_{14-day}$, $\beta _0$ is the independent term, $\beta _1$ is the term that weights the values obtained by our ensemble, and $\beta _i$ is the term that weights the Google mobility variables ($GMD_i\ where\ i = {2, 3, 4, 5, 6, 7}$). GMD variables will be evaluated through t-statistic to figure out if there is a significant relationship between the response variable (14-day CI) and each of the predictors included in the model (ensemble and mobility variables). If so, these variables will be included in the multivariate model.

It is important to note that main assumptions of multivariate regression such as linear relationship between the target variable and the independent variables, normality of all variables, lack of multicollinearity are not met in our case as it is shown in “Evaluation and results” section. Therefore, an operations research approach is proposed to optimize the coefficients of our multivariate model in order to minimize the MAE. Particularly, the Non-Linear Minimization (NLM) procedure^56^, included in R programming software that carries out an iterative minimization procedure is applied to look for optimal coefficients. This method requires a seed to initialize the optimization of the coefficients and three different starting values were analysed: (i) coefficients randomly generated from a uniform distribution from $-10$ to 10, (ii) coefficients with the same weight for each of the independent variables and (iii) coefficient estimates for the multivariate regression model described in Table 9.

## Evaluation and results

This section presents the evaluation of our models for estimating 14-day COVID-19. First, the datasets to perform the experiments are explained. Next, the different univariate ML models and ensemble approach previously explained in “Methods” section for the prediction of the 14-day CI are evaluated. The Google mobility information is then statistically analysed and a PCA is performed to obtain exogenous information to be included in a multivariate model. Finally, the multivariate model with this exogeneous information is evaluated.

### Benchmarking

This section summarizes the datasets used to carry out the experiments. As previously commented, the evaluation is based on the data provided by the Spanish Ministry of Health. They provide several variables for all Spanish regions (19 regions in total). Among them, we may highlight total cases last 24 h, 14-day cumulative incidence and 7-day cumulative incidence. The information is provided by the regional governments that report daily, except on weekends and holidays, to the Spanish Ministry of Health that develops a report with the COVID-19 current situation in Spain. It is important to note that the information is updated backwards when new notifications arrive from previous days, mainly due to delays, error detection, etc. Therefore, we focus on the more stable notification period (i.e. 14-days) as it includes all previous notifications. Particularly, we focus on estimating the 14-day cumulative incidence; i.e. the number of new cases of COVID-19 during 14 days divided by the size of the population at the start of the period.

**Table 1:** Datasets for training and testing ML algorithms.

Dataset name	DS1	DS2
Training period	July, 20–November, 29	July, 20–December, 4
Testing period	November, 30–December, 4	December, 5–December, 18
Testing period trend	Decreasing	Decreasing–increasing

They include different periods with different spatio-temporal characteristics.

Of particular interest is the information from the surveillance system from July, since it changed the way the Spanish Ministry of Health develops the strategy of early detection, monitoring and control of COVID-19. Since then, the count of COVID-19 cases has been kept uniform, with slight changes and updates. Table 1 shows the two different periods under study that are translated into two different datasets. For each period, a train and test datasets have been designed to assess the different trend changes as indicated in the Table 1. Particularly, the first dataset (DS1) includes the information from July 20, 2020 to December 4, 2020. The second dataset (DS2) includes the information from July 20, 2020 to December 18, 2020. In DS1, the models are trained with the information until November 29th, included. The testing, however, is carried out using the data of the week from November 30th to December 4th. In DS2, the models are trained with the information until December 4th, included. The evaluation is carried out with the data from December 5th to December 18th, both included.

**Figure 3:**
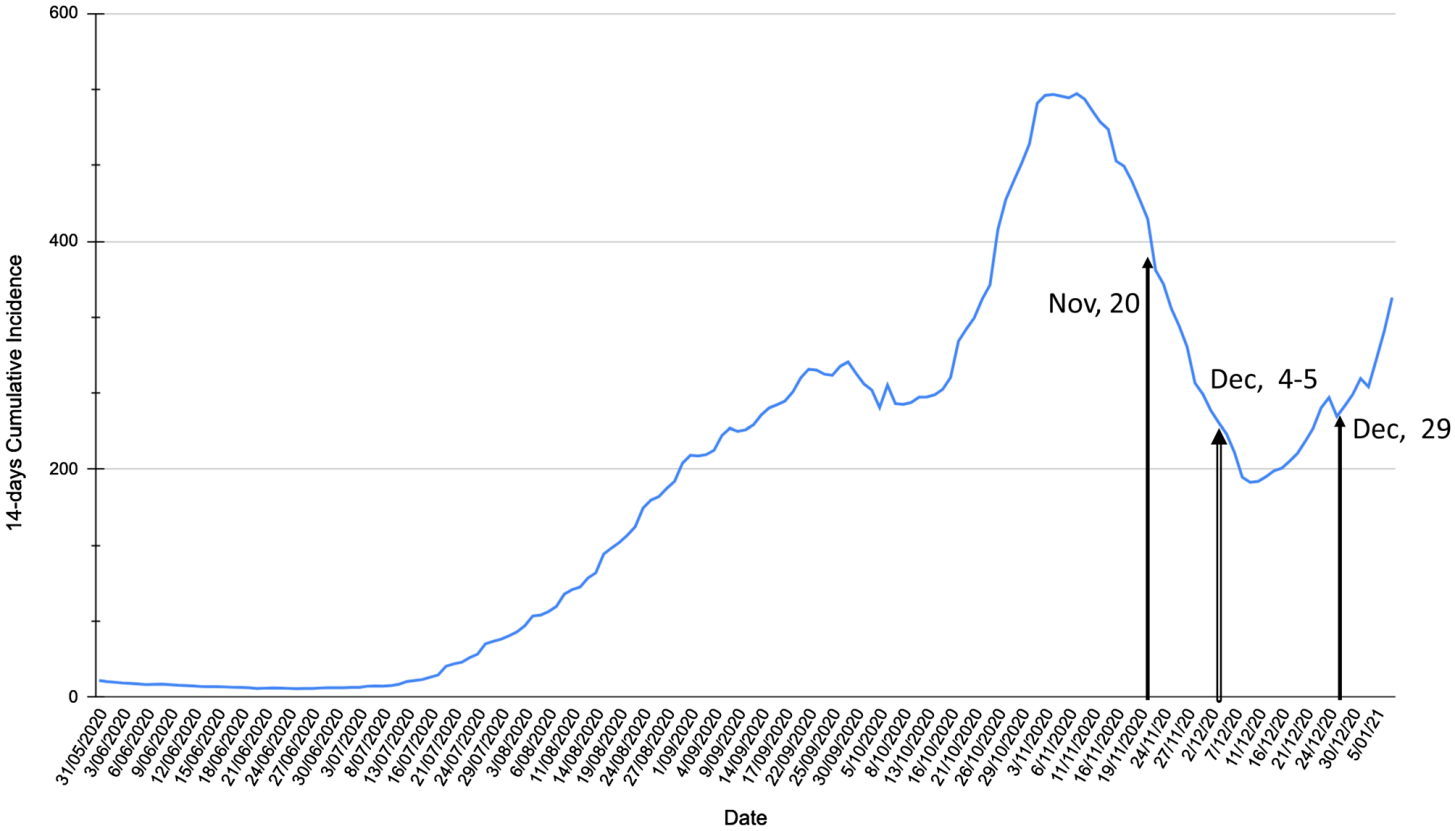
14-day cumulative incidence (CI) in Spain. The evaluation dates are highlighted to let the reader know the trend of 14-day CI at that period.

It is important to note that the 14-day CI was decreasing in the DS1 test period (see Fig. 3). However, the 14-day CI was decreasing at the beginning of the DS2 test period but it suddenly started to increase from December, 11 and beyond. Moreover, DS1 only includes 5 days to predict and DS2 includes 9 days.

Moreover, the metrics used for testing the performance of each model are the coefficient of determination ($R^2$), the root-mean-square error (RMSE) and the mean absolute error (MAE). All of them are calculated using the scikit-learn metrics package^57^. The best possible score for the $R^2$ is 1.0. A constant model that always predicts the expected value of y, regardless of the input features, would get a $R^2$ score of 0.0.

The models obtained have been previously validated and tested using different configurations. For ARIMA-based models, we several (*p*, *d*, *q*) parameters were tested, including (1, 1, 1), (3, 1, 3), (6, 1, 6), (1, 2, 1), (3, 2, 3), (6, 2, 6). For AR-based models, the best performing configurations where those with $p=1,3$ and 6. Finally, Table 2 shows the configurations for GRU and LSTM neural networks that were included in the evaluation. These parameters were empirically determined after several experiments.

**Table 2:** Parameter setup for GRU and LSTM ANNs.

Parameter	LSTM	GRU
Number of input neurons	70	70
Batch size	32	32
Number of epochs	600	600
Learning factor	0.001	0.001
Optimizer	Adam	Adam
Activation function	Hyperbolic tangent	Hyperbolic tangent
Loss function	Mean squared error	Mean squared error
Delay sequence	6	6

Finally, two well-known time series libraries have been included for comparison purposes; i.e., PROPHET^58^ and TPOT^59^. Prophet is a Python-based library developed by Facebook which, according to their authors, “aims at forecasting time series data based on an additive model where non-linear trends are fit with yearly, weekly, and daily seasonality, plus holiday effects. It works best with time series that have strong seasonal effects and several seasons of historical data. Prophet is robust to missing data and shifts in the trend, and typically handles outliers well”. TPOT is also a Python-based automated ML tool that optimizes ML pipelines using genetic programming. TPOT explores many configurations of models and pipelines to find the best one for the target data. The main output of TPOT is a Python code for the best pipeline it has found for your data. These methods have been successfully applied to COVID-19 prediction in different countries such as India, Brazil or UK^60,61^

### 14-day CI estimation

**Table 3:** 14-day CI accuracy prediction for the first dataset.

Model	$\hbox {R}^2$ score	RMSE score	MAE score
GRU	0.96	92.90	91.49
LSTM	0.86	109.91	108.72
AR (1)	> 0.99	37.82	33.01
AR (3)	0.99	6.28	5.61
AR (6)	> 0.99	13.30	13.10
ARIMA (1, 1, 1)	> 0.99	10.67	10.54
ARIMA (3, 1, 3)	0.99	4.48	3.90
ARIMA (6, 1, 6)	0.99	4.96	3.72
ARIMA (1, 2, 1)	> 0.99	16.71	16.04
ARIMA (3, 2, 3)	> 0.99	7.96	7.86
ARIMA (6, 2, 6)	> 0.99	11.08	10.62
Ensemble approach	> 0.99	4.16	3.55
PROPHET	0.99	39.54	36.89
TPOT	0.99	30.94	28.37

Training from July 20, 2020 to November 29, 2020, Prediction from November, 30 to December, 4.

**Table 4:** 14-day CI accuracy prediction for the second dataset.

Model	$R^2$ score	RMSE score	MAE score
GRU	0.59	15.16	11.43
LSTM	0.65	27.18	25.03
AR (1)	0.07	44.79	42.48
AR (3)	0.62	6.84	5.49
AR (6)	0.16	35.11	26.94
ARIMA (1, 1, 1)	0.10	46.21	35.17
ARIMA (3, 1, 3)	0.11	38.50	27.45
ARIMA (6, 1, 6)	0.11	40.41	29.56
ARIMA (1, 2, 1)	0.06	67.44	52.50
ARIMA (3, 2, 3)	0.06	54.76	39.28
ARIMA (6, 2, 6)	0.06	56.33	42.57
Ensemble approach	0.62	6.84	5.49
PROPHET	0.74	20.08	13.21
TPOT	0.01	41.72	31.37

Training from July 20, 2020 to December 4, 2020, Prediction from December, 5 to December, 18.

Tables 3 and 4 show the $R^2$, RMSE and MAE scores for the different ML and statistical models targeted in this study using the evaluation environment previously mentioned in “Benchmarking” section. Let us remind the reader that the main difference between both datasets is the test set. The DS1 develops the prediction in a shorter time series (i.e. 1 week) but with a stable trend (i.e. a decreasing time series). The DS2 develops the prediction in longer time series (i.e. 2 weeks) but with an unstable trend (i.e. increasing and decreasing time series).

**Figure 4:**
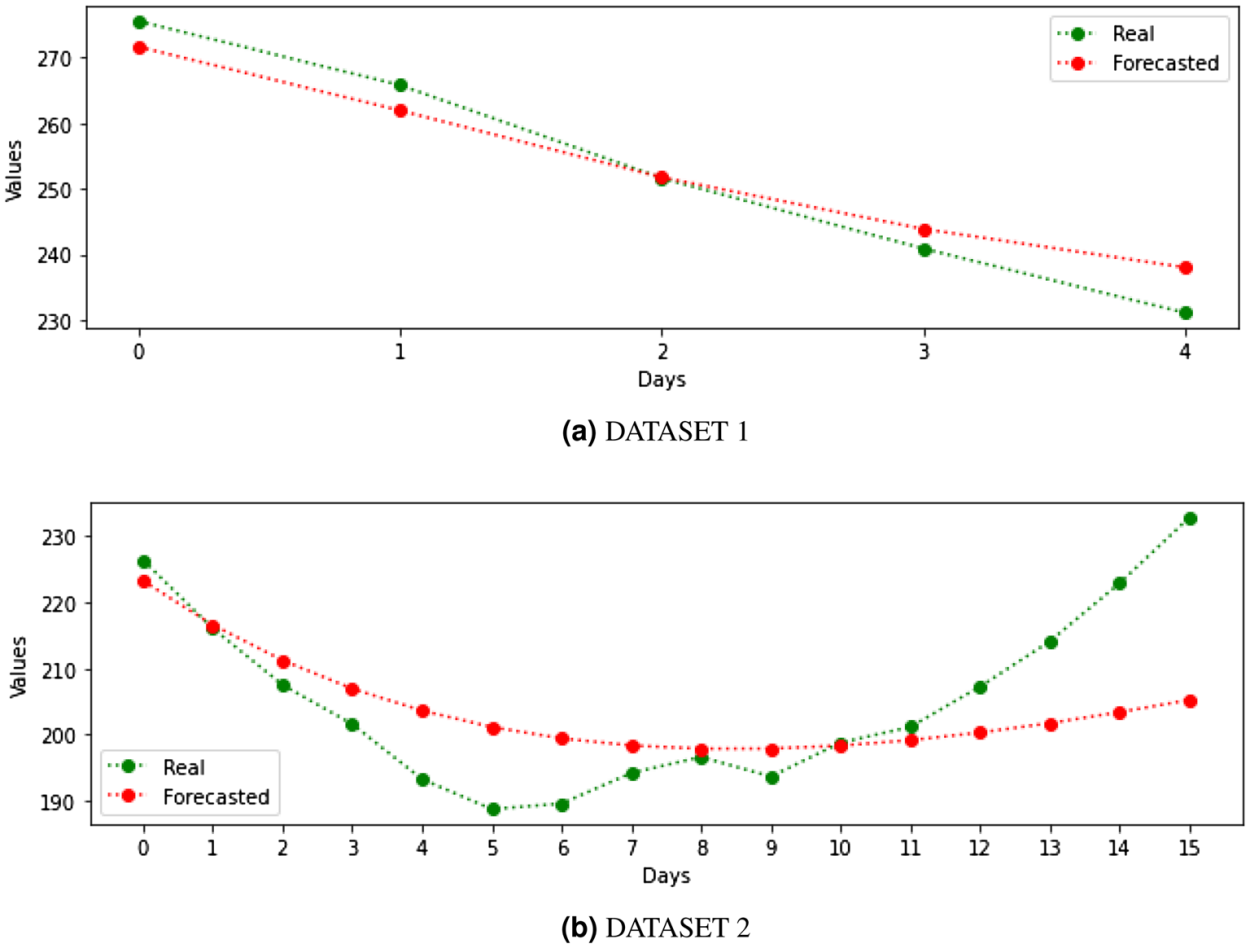
14-day CI accuracy prediction for both datasets.

Table 3 shows the performance of those algorithms when they target the DS1 dataset. In general, artificial neural networks models do not work well for predicting 14-day CI. The dataset includes 1 data item per day, which means a total of data for the largest dataset of up to 109 data items. Therefore, there is not enough information to train the artificial neural network models for a good inference. However, statistical models perform very well in general. The best performing model for the DS1 is the ARIMA with the parameter set up $p=3$, $d=1$, $q=3$, reaching up to 0.99 $R^2$ score, with an RMSE of 4.48 and MAE of 3.90. These results are slightly improved with our ensemble approach, reaching up to 0.99 $R^2$, with an RMSE of 4.16 and MAE of 3.55. Figure 4a shows graphically the actual data and the prediction made by the ensemble for dataset 1.

Table 4 shows the performance of targeted models for the DS2 dataset. The results are significantly worse than those shown in the Table 3. DS2 is more challenging as for the features previously commented (i.e. longer period and unstable trend). Again, our ensemble approach achieves the best performance of all models but, in this case, it only achieves up to 0.62 $R^2$ score, with an RMSE score of 6.84 and MAE score of 5.49. It is important to note that the ensemble approach takes the results of the AR(3) method as the other methods are significantly worse in terms of MAE and RMSE. Moreover, these tests revealed that the prediction of 14-day CI with only historical information performs well for short periods and, above all, clearly marked tendencies. Change in trends due to irregular components are very difficult to predict only using endogenous information and therefore, to improve our forecast for this scenario, we propose the inclusion of an exogenous variable that allows the prediction of these changes in tendency over long periods. Figure 4 shows graphically the actual data and the prediction made by the ensemble for dataset 2.

### Exogeneity evaluation and multivariate model

The inclusion of exogenous variables into the multivariate model requires a preliminary study of the relationship between the 14-day CI and the mobility variables. For that purpose, Spearman’s correlation between 14-day CI and Google mobility variables has been firstly calculated under different scenarios. Table 5 shows Spearman’s correlation between 14-day CI and different lags of the mobility time series.

**Table 5:** Spearman’s correlation between 14-day CI and Google mobility variables for different lags in the mobility time series.

Lags	Retail and recreation	Supermarket and pharmacy	Parks	Public transport	Workplaces	Residential
0	$-$ **0**.**42**	**0**.**28**	$-$ **0**.**59**	0.38	**0**.**23**	**0**.**32**
− 5	$-$0.39	0.21	$-$0.53	0.35	0.14	0.25
− 6	$-$0.38	0.22	$-$0.52	0.36	0.14	0.24
− 7	$-$0.37	0.21	$-$0.51	0.36	0.14	0.22
− 8	$-$0.35	0.21	$-$0.50	0.37	0.14	0.21
− 9	$-$0.34	0.21	$-$0.48	0.37	0.14	0.20
− 10	$-$0.32	0.22	$-$0.47	0.37	0.14	0.19
− 11	$-$0.30	0.22	$-$0.46	0.38	0.13	0.18
− 12	$-$0.28	0.22	$-$0.44	0.39	0.13	0.17
− 13	$-$0.27	0.22	$-$0.43	0.39	0.13	0.15
− 14	$-$0.25	0.23	$-$0.42	**0**.**40**	0.13	0.13

The analysis in Table 5 indicates that most mobility variables have a relevant correlation with 14-day CI, especially retail and recreation, parks and public transport. Interestingly, leisure-related mobility variables, i.e. retail and recreation and parks, have a negative correlation with CI while non-leisure mobility variables have a positive correlation. Additionally, it is worth highlighting that the two situations are distinguished. If the correlation between 14-day CI and a mobility variable (in absolute value) grows as the lags of the exogenous variable increases, past values of the mobility variable have a more significant association with current cumulative incidence than recent ones. In contrast, if correlation decreases as the number of lags augments, the corresponding mobility variable might be considered either not significantly associated with 14-day CI or more significantly related with 14-day CI for recent values of the mobility variable. This underscores a pragmatic limitation of univariate models, in that available exogenous variables cannot be used to forecast changes in 14-day CI curve trend such as an uptick in new coronavirus cases.

Nevertheless, in practice, the establishment of causal statements between series of observations is not straightforward. Our interest is to examine whether mobility time series helps to predict future values of 14-day CI, controlling for lags. Table 6 reports Granger causality test outcomes for different lag orders analysing whether past values of mobility variables provide additional information about 14-day CI beyond past values of 14-day CI.

**Table 6:** Granger causality testing mobility variables predictive of 14-day CI for different lag orders.

Lags	Retail and recreation	Supermarket and pharmacy	Parks	Public transport	Workplaces	Residential
5	0.03	0.72	< 0.01	< 0.01	0.52	0.16
6	0.01	0.66	0.01	< 0.01	0.17	0.22
7	0.01	0.70	0.02	< 0.01	0.18	0.28
8	0.03	0.61	0.08	< 0.01	0.17	0.37
9	< 0.01	0.49	0.17	< 0.01	0.13	0.14
10	0.02	0.78	< 0.01	< 0.01	0.19	0.30
11	< 0.01	0.32	0.01	< 0.01	0.31	0.32
12	0.01	0.35	< 0.01	< 0.01	0.35	0.29
13	< 0.01	0.15	0.01	< 0.01	0.19	0.19
14	< 0.01	0.04	0.01	< 0.01	0.21	0.01

From the results in Table 6, the effect of lags of mobility variables retail and recreation, parks and public transport on 14-day CI is highly significant whatever the number of lags is. The stationarity of the variables was previously checked using the Augmented Dickey-Fuller test via the adf.test function in R. Bearing this in mind, according to WHO, the incubation period of COVID-19 is on average 5–6 days but can be as long as 14 days, lags have been considered varying from 5 to 14 days. However, it is important to note that too few lags can lead to a biased test due to residual autocorrelation whereas with too many, null hypothesis might be incorrectly rejected because of spurious correlation. Therefore, the number of lags that need to be chosen reaching is a tradeoff between bias and power. Then, it can be concluded that these three mobility variables are predictive of future cumulative incidence figures.

Reciprocally, Granger causality tests analysing whether 14-day CI values help to predict future values of mobility variables have been run and corresponding p-values are shown in Table 7. According to these results, 14-day CI is highly significant on retail and recreation for every lag order and, in general, for the rest of the mobility variables from a lag length of 8. In other words, 14-day CI is predictive of mobility variables in a period of a week from current values. This finding is consistent regarding the incubation period; however, these results should be cautiously interpreted. An increase in new coronavirus cases is bound to force government intervention and the application of measures aimed at restricting citizens mobility. Likewise, a decline of the 14-day CI curve would lead to social relaxation, which would be translated into an increase in mobility.

**Table 7:** Granger causality testing 14-day CI predictive of mobility variables for different lag orders.

Lags	Retail and recreation	Supermarket and pharmacy	Parks	Public transport	Workplaces	Residential
5	< 0.01	0.20	0.38	0.26	0.05	0.25
6	< 0.01	0.35	0.10	0.17	0.31	0.08
7	< 0.01	0.01	0.21	0.03	< 0.01	< 0.01
8	< 0.01	0.02	0.04	< 0.01	< 0.01	< 0.01
9	0.01	< 0.01	0.12	< 0.01	< 0.01	< 0.01
10	0.01	< 0.01	0.13	< 0.01	< 0.01	< 0.01
11	0.03	0.01	0.12	< 0.01	< 0.01	< 0.01
12	0.05	0.01	0.16	< 0.01	< 0.01	< 0.01
13	0.02	0.02	0.17	< 0.01	< 0.01	< 0.01
14	0.03	0.04	0.30	< 0.01	< 0.01	< 0.01

As a result, reverse or bidirectional causation may be present in our problem. Therefore, we cannot conclude that mobility variables potentially cause future values of 14-day CI. Moreover, government containment measures in mobility, nightclubs or bars and other factors such as social alarm also involve changes in 14-day CI trends and thus, there may be latent confounders that are correlated with 14-day CI underlying the true cause of the evolution of new coronavirus cases. Hence, making a strong causal statement is hard, however, our intention was less ambitious targeted at shedding light on what mobility variables are useful for predicting 14-day CI.

Based on this preliminary study, the results obtained by our ensemble approach, retail and recreation, parks and public transport time series will be used hereafter as explanatory variables to develop a multivariate model where 14-day CI is the response variable. Because the average incubation period of COVID-19 outlined by the WHO lasts a minimum of 5 days, selected mobility variables will be considered 5 periods lagged. Furthermore, Google mobility variables will be standardised and rescaled to the last three days of 14-day CI before predictions are made in order to provide meaningful information to the model.

**Figure 5:**
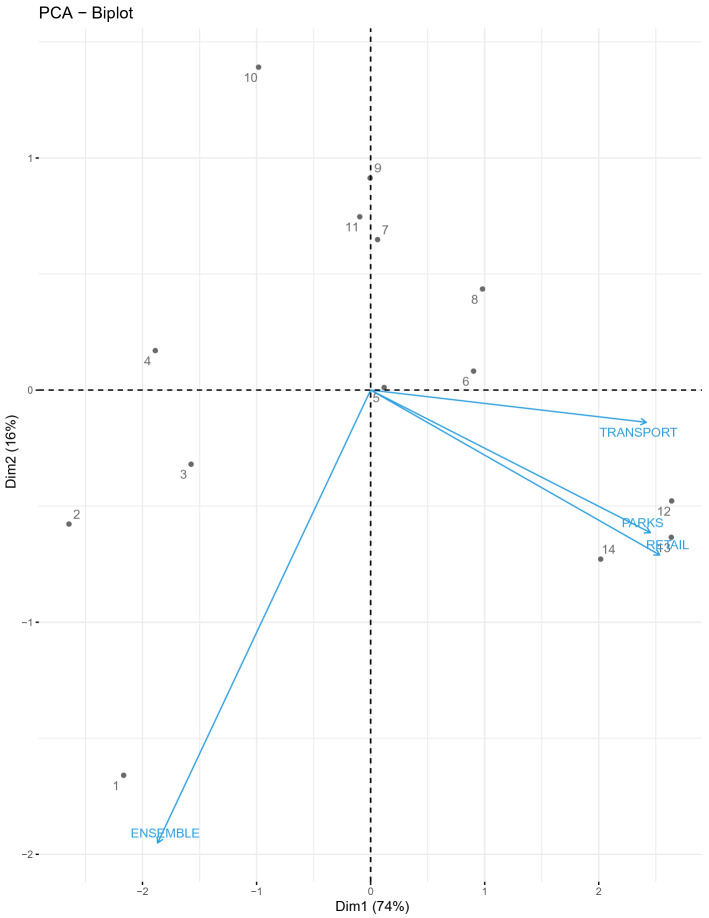
PCA to ensemble approach and mobility variables. Positively correlated variables point to the same side of the plot. Negatively correlated variables point to opposite sides of the graph.

Finally, a principal component analysis (PCA) is computed considering these variables. Table 8 indicates that two components would preserve more than 87% of the total variance in the original data. In other words, two components explain more than 87% of the information provided by the exogenous variables. Figure 5 graphically illustrates that mobility variables are clearly differentiated from the ensemble approach in the PCA analysis. Thus, mobility variables would provide additional information to the proposed multivariate model.

**Table 8:** Eigenvalues and proportion of variance (i.e. information) explained by each component in the PCA.

Number of components	Eigenvalues	Proportion of variance (%)	Cumulative proportion (%)
1	2.91	72.83	72.83
2	0.597	14.93	87.76
3	0.391	9.77	97.52
4	0.099	2.48	100

**Table 9:** Multivariate regression for DS2 training period.

Coefficients	Estimate	Std. Error	*p*-value
$\beta _0$ (Independent)	− 110.59	259.76	0.68
$\beta _1$ (Ensemble)	1.31	0.26	< 0.01
$\beta _2$ (Retail and recreation)	1.00	0.59	0.13
$\beta _3$ (Parks)	− 0.20	1.29	0.88
$\beta _4$ (Public transport)	− 0.60	0.63	0.37

$$R^2=0,79$$, *p*-value $$<0.01$$.

In particular, this paper includes an optimization model aimed at improving forecasts in 14-day CI time series which uses multivariate regression as starting point. Table 9 shows the regression outcomes obtained for DS2 training period. The coefficient estimates and standard errors are calculated. The p-value corresponding to the t-statistic of each coefficient indicates if there is a significant relationship between the response variable (14-day CI) and each of the predictors included in the model (ensemble and mobility variables). Table 10 shows the results obtained by the NLM method for the different seed values previously described in “Methods” section, i.e. the MAE and the number of iterations performed by the procedure in each case. It is important to highlight that when the seed of NLM is the coefficients randomly generated from a uniform distribution from -10 to 10, the NLM algorithm is executed 10 times and the MAE and number of iterations in Table 10 are calculated as the average over 10 simulation runs. As can be seen, the best result is reached by performing 36 iterations of the algorithm, it returns a MAE of 3.77 and it is achieved when the NLM procedure uses the multivariate regression model as seed.

**Table 10:** MAE achieved and iterations performed by NLM procedure using different seeds.

Seed	Avg. of 10 random runs	Weighted equally	Multivariate regression model
MAE	4.66	4.06	3.77
NLM iterations	50	46	36

**Figure 6:**
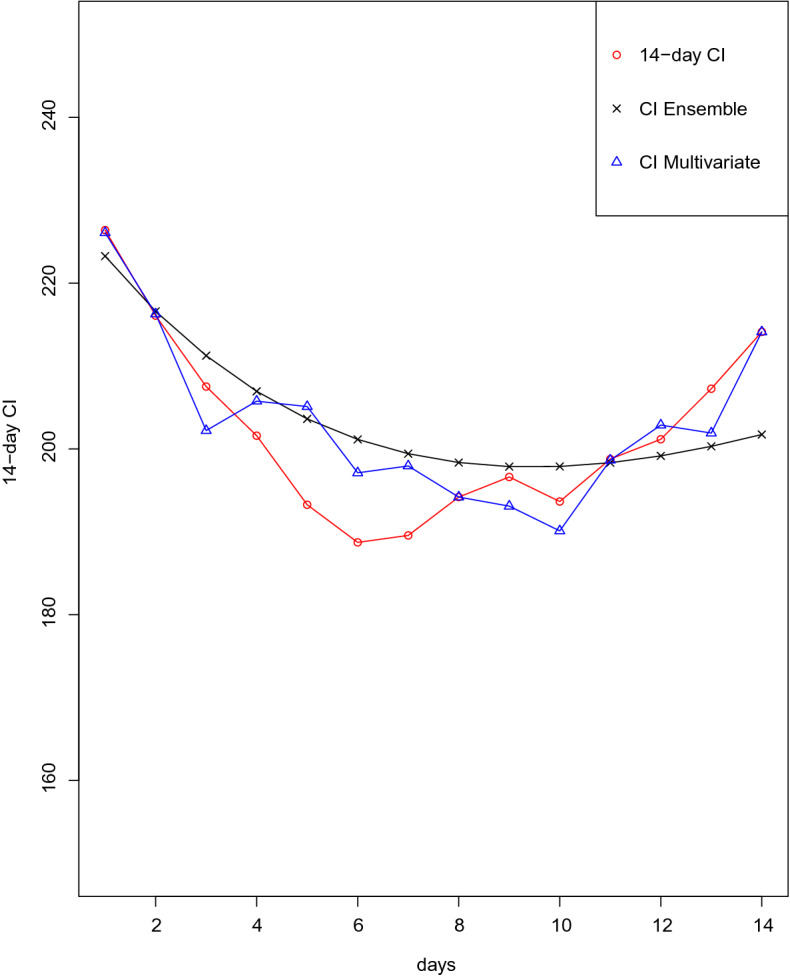
14-day CI accuracy prediction for different estimated models.

Once the MAE has been minimized, Table 11 presents 14-day CI predictions for an evaluation period from 5th to 18th of December using the multivariate model with the optimal coefficient values obtained by NLM for the minimum MAE. It is important to remark that if exogenous variables are not extended, 14-day CI forecasts are restricted to a five-period prediction horizon. Nonetheless, forecasts in the evaluation period have been obtained using the observed past values of the mobility variables. This approach might not be realistic, but the purpose of the study is to validate the performance of the model using mobility data regarding other ML methods not including this exogenous information. To assess the accuracy of the model, the mean absolute error is measured and a comparison is made with regard to predictions given by the univariate strategy in the ensemble approach. In addition, Fig. 6 shows true 14-day CI curve and the ensemble approach and multivariate predicted values throughout the forecast horizon. It is noteworthy that the multivariate model substantially outperforms the ensemble approach. The results also suggest that both models produce reasonably good estimates, but the multivariate model tracks better changing trends in 14-day CI.

To conclude, it is interesting to note that predictions made from 16th to 18th of December (labeled by 12, 13, 14 in Fig. 5), when a new uptick in coronavirus infections and hospitalizations began, are located in the exogenous area of the PCA graphics meaning that for these values mobility variables have a higher impact. Again, these results evidence that exogenous variables offer valuable information to cope with trend changes in the 14-day CI curve and justifies the use of a multivariate model.

**Table 11:** 14-day CI accuracy prediction for ensemble approach (EA) and NLM methog (NLM).

DATE	14-day CI	CI Ensemble	CI NLM	$\hbox {MAE}_{EA}$	$\hbox {MAE}_{NLM}$
December 5	226.39	226.08	225.10	3.14	0.31
December 6	216.07	216.28	214.58	1.83	0.26
December 7	207.52	202.21	204.94	2.46	1.94
December 8	201.59	205.76	204.93	3.18	2.50
December 9	193.26	205.11	202.78	4.62	4.37
December 10	188.72	197.11	197.34	5.92	5.04
December 11	189.56	197.94	195.49	6.48	5.52
December 12	194.19	194.19	193.76	6.19	4.83
December 13	196.61	193.09	191.53	5.64	4.68
December 14	193.65	190.11	188.13	5.50	4.57
December 15	198.77	198.64	195.77	5.04	4.16
December 16	201.16	202.87	201.91	4.79	3.96
December 17	207.26	201.91	202.32	4.96	4.07
December 18	214.12	214.11	210.12	5.49	3.78

Training from July 20, 2020 to December 4, 2020, Prediction from December, 5 to December, 18.

## Discussion

The use of a regression model entails the acceptance of assumptions that may be questionable at best in the context of time series data. Methodologically, this approach is flawed mainly because accuracy may be seriously affected in the presence of autocorrelation. Furthermore, difficulties in data collection due to discrepancies in regional notifications and differences on COVID-19 medical tests carried out are added to statistical problems, which are compounded when data include measurement error. In view of the foregoing, this multivariate technique cannot be used as an inference method. However, the use of an operation research optimization method such as NLM implementing the regression coefficients as a seed improves the solution obtained by the univariate model. Evidently, this option has its own drawbacks such as the problem of falling in local optima or the setting of good initial values for the solver.

The ensemble approach rendered a smoother curve that could not detect trend changes. Indeed, the results provided by the ensemble approach reinforce the need for monitoring models that can also detect changes in trend with some foresight. Accordingly, despite the potential limitations mentioned above, the proposed multivariate approach can be gainfully used for predicting possible upticks in COVID-19 cases at least in a short-term period. Therefore, the inclusion of the two models within a decision support system provides us with a positive result that covers the different types of data behavior, both when the trend is constant and in the changes of trend. In this system, depending on the error produced by each model when introducing a new value to predict, it will be selected either the ensemble approach or the multivariate approach.

## Conclusions and future work

COVID-19 has caused one of the biggest crises in our recent history. Most countries have developed monitoring systems based on pandemic evolution indicators to trigger social distancing measures whenever significant increases in infections are detected. Data analysis can help forecast the short- and medium-term evolution of the pandemic and thus help policymakers in their decision making. In this paper, we have analysed the evolution of the 14-day cumulative incidence in Spain from the beginning of the second wave of COVID-19 until January 2021, where several trend changes (also called waves) occurred. We have proposed a set of statistical and ML models to achieve maximum performance, reaching very good results for short and stable periods. However, the 14-day CI is affected by irregular components which are very challenging scenarios for traditional models using only historical information. Therefore, the mobility data provided by Google as a consequence of the COVID-19 outbreak are fed into our models as exogenous information to predict these irregular components. Our results reveal that this information improves the prediction of this unstable scenario, providing an MAE of up to 1.08 on average.

Data fusion between socio-economic and endogenous variables is still at a relatively early stage, and we acknowledge that we have tested a relatively simple variant of a multivariate model. But, with many other types of multivariate models and data such as vaccination figures yet to be explored, this field seems to offer a promising and potentially fruitful area of research. Moreover, this approach can be followed at the international level to predict changes in trends and coordinate the pandemic globally.
